# Angiopoietin-Like 4 Is Over-Expressed in Rheumatoid Arthritis Patients: Association with Pathological Bone Resorption

**DOI:** 10.1371/journal.pone.0109524

**Published:** 2014-10-07

**Authors:** Catherine Swales, Nicholas A. Athanasou, Helen J. Knowles

**Affiliations:** 1 Botnar Research Centre, Nuffield Department of Orthopaedics Rheumatology and Musculoskeletal Sciences, University of Oxford, Oxford, United Kingdom; 2 Pathology Department, Nuffield Orthopaedic Centre, University of Oxford, Oxford, United Kingdom; Université Jean Monnet, France

## Abstract

**Introduction:**

Osteoclasts are responsible for the bone loss associated with rheumatoid arthritis (RA). The secreted adipokine angiopoietin-like 4 (ANGPTL4) specifically increases osteoclast-mediated bone resorption. We have investigated expression of ANGPTL4 and its regulatory transcription factor, hypoxia-inducible factor-1 alpha (HIF-1α), in osteoclasts and other cells within rheumatoid synovium. We have also examined whether circulating levels of ANGPTL4 differ in RA patients compared with that in normal controls or patients with osteoarthritis (OA).

**Results:**

Immunohistochemical analysis revealed that bone-apposing osteoclasts within the rheumatoid synovium express both ANGPTL4 and HIF-1α. ANGPTL4 was also strongly expressed in synovial lining cells, endothelial cells, stromal cells, CD68+ macrophages and plasma cells within RA synovium. Little ANGPTL4 was evident in normal synovial tissue. This reflected the over-expression of HIF-1α in rheumatoid versus normal synovial tissue. The concentration of ANGPTL4 was higher in both the serum and the synovial fluid of RA patients than in patients with OA or normal controls. High serum ANGPTL4 associated with elevated levels of the serum marker of bone resorption, receptor activator for nuclear factor κB ligand (RANKL).

**Conclusions:**

Over-expression of ANGPTL4 in multiple cell types within the rheumatoid synovium potentially provides a local pool of ANGPTL4 to stimulate osteoclast-mediated bone resorption in RA. Additionally, correlation of high serum ANGPTL4 with circulating RANKL suggests that ANGPTL4 may represent a novel marker for bone destruction in RA.

## Introduction

Rheumatoid arthritis (RA) is a chronic inflammatory disease characterised by erosion of articular cartilage and subchondral bone, leading to joint destruction, pain and loss of function. Bone loss in RA, comprising both juxta-articular bone erosions and systemic osteoporosis, is associated with progressive disability and is predictive of poor prognosis [1], [2]. Both types of bone loss are mediated by osteoclasts [3], [4], multinucleated cells specialised to carry out lacunar bone resorption. Inhibition of osteoclast activity effectively inhibits both the formation of new focal bone erosions and systemic bone loss [5], [6].

In contrast to normal synovial tissue the rheumatoid synovium is hypoxic. Low tissue oxygen levels are also a poor prognostic indicator in RA [7], [8]. Synovial lining cells, endothelial cells, fibroblasts, lymphocytes, plasma cells and CD68+ macrophages within the RA synovium express the hypoxia-inducible transcription factor, HIF [9]–[11]. HIF is a heterodimer comprising a hypoxia-inducible alpha subunit and a constitutively expressed beta subunit. Hypoxic accumulation and activation of HIF-alpha allows transcription of genes involved in processes including angiogenesis, inflammation, apoptosis and regulation of immune function [12].

HIF has not been described in osteoclasts in RA, although HIF-1α-positive osteoclasts are present in other osteoclast-rich pathologies such as the primary bone tumour Giant Cell Tumour of Bone [13]. Transient hypoxia increases osteoclast formation [14], [15] by stimulating the fusion and differentiation of circulating CD14+ mononuclear precursors or synovial macrophages in the presence of macrophage-colony stimulating factor (M-CSF) and RANKL [16], [17]. Hypoxia also causes increased bone resorption, via a HIF-1α-dependent mechanism to enhance osteoclast activity by increasing production of ATP under hypoxic conditions [14], [18].

Angiopoietin-like 4 (ANGPTL4) is a HIF-1α-inducible pro-angiogenic adipokine [19], [20]. It is over-expressed in the hypoxic, perinecrotic regions of solid tumours [19] and induced by hypoxia in multiple cell types including endothelial cells [19], articular chondrocytes [21], osteoclasts, osteoblasts [13] and fibroblast-like RA synoviocytes [22]. We have previously demonstrated that ANGPTL4 directly stimulates osteoclast activity and promotes bone resorption *in* *vitro* via a mechanism independent of RANKL [13].

This study investigates the over-expression of both ANGPTL4 and HIF-1α in RA, with specific reference to their expression in osteoclasts. We also assess correlations between the serum concentration of ANGPTL4 and markers of pathological bone resorption in RA.

## Materials and Methods

### Patients

RA patients were recruited through the rheumatology clinics at the Nuffield Orthopaedic Centre and serum samples were stored at −80°C until analysis. Synovial fluid was aspirated from the knee joints of patients with RA and non-inflammatory OA as previously described [23], OA being defined as non-inflammatory when neutrophils comprised <25% of the total synovial fluid cell population sampled. Serum from OA and injury repair patients and all formalin-fixed, paraffin-embedded tissue sections were obtained from the Oxford Musculoskeletal BioBank. RA was defined according to the 1987 American College of Rheumatology criteria and OA was diagnosed on clinical, radiographic and histologic criteria. Samples and/or data obtained were collected with informed written donor consent in full compliance with national and institutional ethical requirements, the United Kingdom Human Tissue Act and the Declaration of Helsinki. This study was approved under Oxford Musculoskeletal BioBank ethics (HTA Licence 12217, Oxfordshire Research Ethics Committee C, 09/H0606/11). RA and OA synovial fluid samples were collected with approval of the Oxford Central University Research Ethics Committee, 06/Q1606/139.

### Immunohistochemistry and immunofluorescence

Wax-embedded samples of rheumatoid synovial tissue were sectioned and H&E stained. Antigen retrieval of deparaffinised sections was performed by microwaving in 1 mM EDTA (pH 8). Sections were exposed to anti-ANGPTL4 (H-200; Santa Cruz Biotechnology), anti-cathepsin K (clone 182-12G5, Calbiochem), anti-CD20 (clone L26; Dako), anti-CD68 (clone KP1; Dako), anti-HIF-1α (clone 54; BD Biosciences) or a serum control. For standard immunohistochemistry, staining was visualized with the VECTASTAIN Elite ABC Kit (Vector Laboratories). For immunofluorescence, secondary antibodies were DyLight 488 or 594 conjugates (Thermo Scientific). Image acquisition was performed using a Zeiss AxioImager MI microscope, AxioCam HRC camera and AxioVision software.

Osteoclasts in tissue sections were considered as large, multinucleated cells containing >3 nuclei; a widely accepted identification criterion in RA [23]–[26]. Osteoclast identification was additionally confirmed by staining for cathepsin K; an enzyme expressed at high levels in osteoclasts [27], [28] and a validated osteoclast marker in RA [29], [30].

### Cell culture

Peripheral blood mononuclear cells were isolated from the blood of patients with RA using Histopaque (Sigma). CD14+ monocytes were selected (AutoMACS cell separator; Miltenyi Biotech), seeded into α-MEM (without ribonucleosides/deoxyribonucleosides, containing 10% FBS, 2 mM L-glutamine, 50 IU/ml penicillin and 50 µg/ml streptomycin sulphate) and differentiated into osteoclasts by supplementing with M-CSF (25 ng/ml) and RANKL (50 ng/ml) every 3–4 days for 12–16 days. Hypoxic exposure was at 2% O_2_, 5% CO_2_, balance N_2_ for 24 h (MiniGalaxy incubator, RS Biotech).

### Real-time PCR

Total RNA was extracted in TRI reagent (Sigma), treated with DNase I and reverse-transcribed (SuperScript VILO cDNA Synthesis Kit, Invitrogen). Real-time PCR was performed using Express SYBR GreenER qPCR Supermix Universal (Invitrogen) and predesigned intron-spanning QuantiTect primers (Qiagen) on a RotorGene RG-3000 (Corbett Research UK). Comparative quantification was performed, with target gene expression normalized to β-actin (ACTB).

### ELISAs

Serum was analysed from 19 RA patients (age 62.1±13.7, 21.1% male, 78.9% female; clinical and serological characteristics are described in Table 1) and age- and sex-matched OA patients (n = 30; age 61.8±8.1, 20% male, 80% female) and normal controls (n = 30 surgical injury repair patients; age 61.0±7.8, 20% male, 80% female). Synovial fluid from a separate cohort of 8 RA patients and 7 non-inflammatory OA patients was digested with 2 mg/ml hyaluronidase prior to assay [23]. ANGPTL4 (R&D Systems), C-reactive protein (CRP) (Invitrogen) and RANKL (Peprotech) were assayed according to the manufacturer’s instructions.

**Table 1:** Clinical characteristics of RA patients in serum cohort.

Patient no.	Sex (M/F)	Age (yrs)	Seropositive (Y/N)	DAS score	CRP (µg/ml)
R1	F	70	Y	3.52	9.15
R2	F	72	Y	1.4	5.26
R3	F	87	Y	2.71	1.17
R4	F	67	Y	2.9	10.97
R5	F	45	Y	4.5	5.48
R6	F	69	N	2.14	2.39
R7	F	73	Y	2.88	0.42
R8	M	64	-	2.5	0.5
R9	M	40	Y	7.35	13.46
R10	F	48	Y	3.5	0.31
R11	F	59	Y	2.03	4.24
R12	F	34	Y	6.2	36.67
R13	F	66	Y	2	8.12
R14	F	44	N	3.32	0.79
R15	M	63	-	1.81	1.35
R16	M	76	Y	1.4	1.68
R17	F	65	Y	3.05	15.0
R18	F	70	-	-	4.36
R19	F	68	Y	4.1	8.7

DAS  =  disease activity score. CRP  =  C-reactive protein.

### Statistics

For experiments with 2 conditions a Wilcoxon matched-pairs or Mann-Whitney test was used as appropriate. Statistics for serum data were performed using the Pearson correlation coefficient or Fisher’s exact test. Results were calculated in GraphPad Prism 6 and considered significant at p<0.05.

## Results

### ANGPTL4 is strongly expressed by osteoclasts in RA

**Figure 1:**
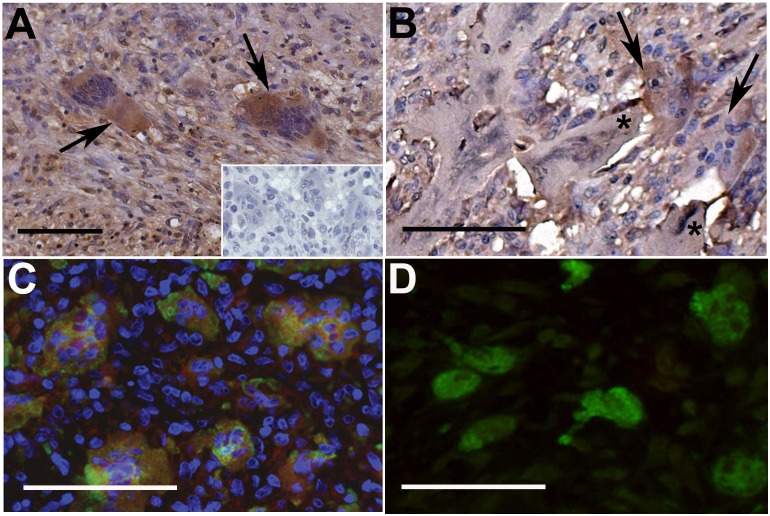
ANGPTL4 is expressed by osteoclasts in RA. (A) ANGPTL4 is strongly expressed by osteoclasts (arrows) in the RA synovium. Inset: Representative isotype control image. (B) Resorbing osteoclasts adjacent to bone (starred) express ANGPTL4. (C) ANGPTL4 (red) expression co-localises with cathepsin K-positive (green) osteoclasts. Representative images from n = 3 cases. (D) Cathepsin K-positive (green) osteoclasts in the OA synovium do not express detectable ANGPTL4 (red). All scale bars represent 100 µM.

Rheumatoid synovial sections were screened for the presence of multi-nucleated cells (>3 nuclei) morphologically characteristic of osteoclasts. Numerous synovial osteoclasts were identified in 3/8 cases. In each instance, immunohistochemical analysis of the hyperplastic synovial tissue (Figure 1A) and synovium-bone interface (Figure 1B) revealed strong expression of ANGPTL4 in multi-nucleated cells that were confirmed as osteoclasts by double staining with cathepsin K (Figure 1C). Although other synovial cells were also ANGPTL4-positive, over 95% of osteoclasts expressed ANGPTL4 more strongly than the surrounding synovial cells in any given section. Scattered osteoclasts present in OA synovial sections were smaller (fewer nuclei) than those in RA and did not express immuno-detectable ANGPTL4 as revealed by either immunohistochemistry or immunofluorescence (Figure 1D).

### RA osteoclasts express HIF-1α protein

**Figure 2:**
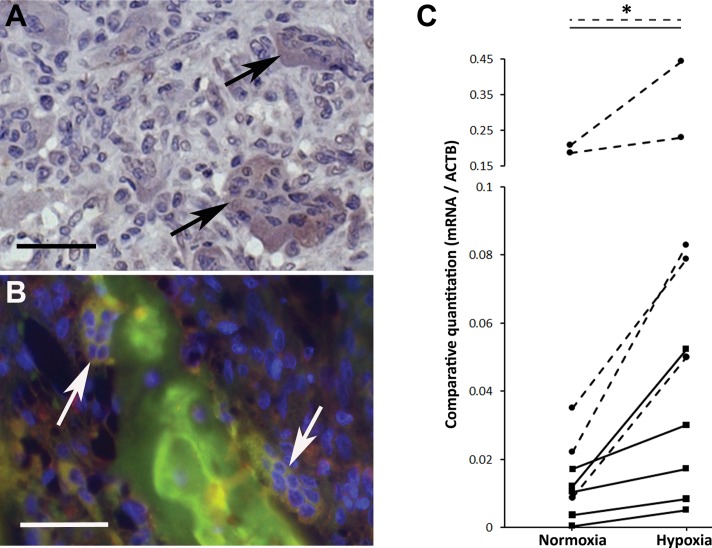
HIF-1α is expressed by osteoclasts in RA. (A) HIF-1α expression by osteoclasts in the RA synovium (arrows). Representative image from n = 3 cases. (B) HIF-1α (green) and ANGPTL4 (red) expression co-localises in 2 bone-apposing osteoclasts (arrows). All scale bars represent 100 µM. (C) ANGPTL4 (solid lines) and SLC2A1 (Glut-1; dashed lines) mRNA expression in monocyte-derived osteoclasts from RA patients following 24 h exposure to normoxia or hypoxia (2% O_2_). Hypoxic fold-change in mRNA expression; *, p<0.05.

Cytoplasmic expression of HIF-1α was observed in 22–50% of osteoclasts in any given rheumatoid synovial tissue section (Figure 2A). Where resorbing osteoclasts were visible at the synovium-bone interface, co-localised expression of HIF-1α and ANGPTL4 was also evident (Figure 2B). Osteoclasts derived from the circulating CD14+ monocytes of RA patients exhibited increased expression of ANGPTL4 mRNA following exposure to hypoxia (Figure 2C; mean induction 5.5-fold, range 1.7- to 17.2-fold). Expression of other HIF target genes was also increased (e.g. SLC2A1; mean induction 3.0-fold, range 1.2- to 5.6-fold).

### ANGPTL4 is over-expressed in synovial tissue in RA

**Figure 3:**
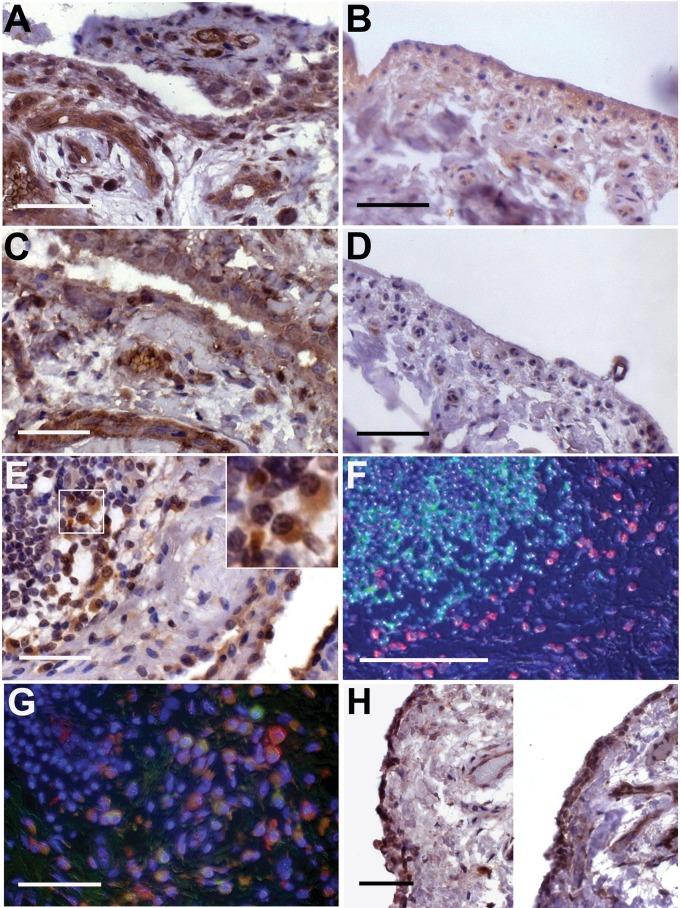
ANGPTL4 and HIF-1α are expressed in RA synovial tissue. (A) The RA synovium is strongly positive for ANGPTL4, as are adjacent blood vessels and surrounding stromal cells; (B) normal synovium shows weak, heterogeneous expression of ANGPTL4. (C) RA synovium is strongly positive for HIF-1α, as are adjacent blood vessels; (D) normal synovium generally does not express HIF-1α. (E) Synovial lining cells and stromal cells adjacent to lymphoid aggregates express ANGPTL4. Inset: ANGPTL4-positive plasma cells. (F) ANGPTL4 (red) is not expressed by B cells (CD20, green). (G) ANGPTL4 (red) is expressed by CD68-positive (green) macrophages adjacent to a lymphoid aggregate. (H) The OA synovium expresses elevated levels of both ANGPTL4 (left panel) and HIF-1α (right panel) compared with the normal synovium. Scale bars (A–F, H) represent 100 µM, scale bar (G) represents 50 µM.

As ANGPTL4 reactivity was observed in the synoviocytes adjacent to osteoclasts, we also studied ANGPTL4 expression in 5 additional osteoclast-negative cases of RA. ANGPTL4 was strongly expressed by the synovial lining cells, endothelial cells and stromal fibroblasts within RA synovial tissue (Figure 3A), compared with general low-level expression in normal synovium (Figure 3B). The expression pattern of ANGPTL4 was similar to that for HIF-1α in RA synovial sections (Figure 3C). HIF-1α was rarely detected in normal synovium (Figure 3D).

ANGPTL4 was also strongly expressed in the immediate vicinity of lymphoid aggregates in RA, in cells morphologically characteristic of macrophages and plasma cells (Figure 3E). Immunofluorescence confirmed that ANGPTL4 was not expressed by B cells (Figure 3F) or T cells (data not shown), but was expressed by CD68-positive macrophages (Figure 3G).

Both ANGPTL4 (Figure 3H, left panel) and HIF-1α (Figure 3H, right panel) were over-expressed in OA synovial tissue in comparison with the normal synovium, although not to the same degree (number of positive cells per field of view) as in RA synovial tissue.

### ANGPTL4 is elevated in the serum and synovial fluid of RA patients

**Figure 4:**
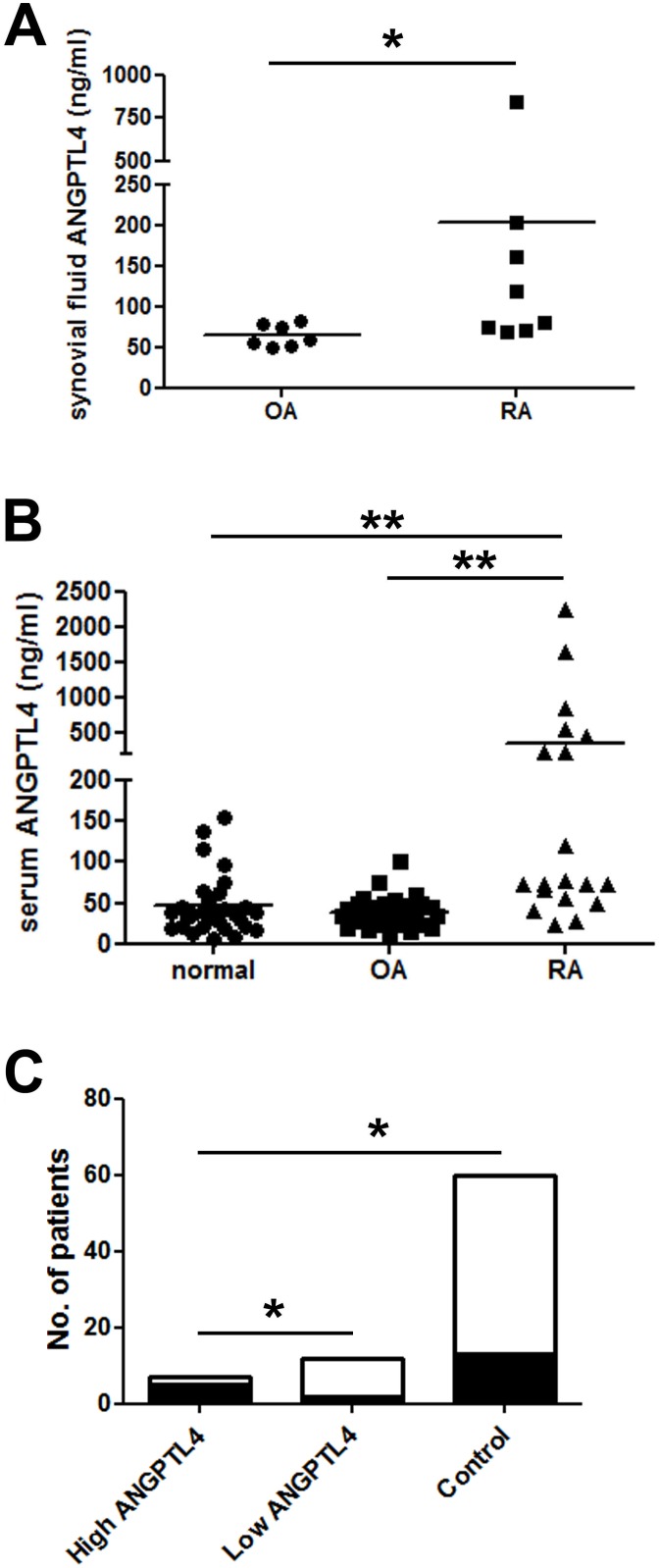
Serum and synovial fluid ANGPTL4 concentrations are elevated in RA. (A) Synovial fluid from RA patients contains more ANGPTL4 (203.3±264.8 ng/ml, range 68.7–847.3 ng/ml) than that from patients with non-inflammatory OA (64.4±13.6 ng/ml, range 50.4–82.2 ng/ml). *, p<0.05. (B) Serum from RA patients contains more ANGPTL4 (363.4±138.7 ng/ml, range 24.0–2235.0) than that from OA patients (39.0±3.4 ng/ml, range 11.9–100.5 ng/ml) or normal controls (45.8±6.7 ng/ml, range 6.5–154.7 ng/ml). **, p<0.01. (C) Serum from ‘high ANGPTL4’ RA patients is more likely to have detectable RANKL (black shading; RANKL-positive) than either serum from ‘low ANGPTL4’ RA patients or controls. White shading; RANKL-negative. *, p<0.05.

Given the striking over-expression of ANGPTL4 by multiple cell types within RA synovial tissue, we next assessed whether concentrations of secreted ANGPTL4 were also elevated in RA synovial fluid. ANGPTL4 was measured in synovial fluid aspirated from the knee joints of patients with RA and non-inflammatory OA controls. The ANGPTL4 concentration was significantly higher in the clarified synovial fluid of the RA population (Figure 4A).

Due to low patient numbers in the synovial fluid cohort, we expanded the study to look at serum concentrations of ANGPTL4. ANGPTL4 levels were significantly higher in the serum of patients with RA than in either OA patients or normal controls (Figure 4B). Within the RA population, serum ANGPTL4 positively correlated with age (r = 0.460, p<0.048).

The RA cohort comprised 2 sub-groups based on the serum level of ANGPTL4; ‘low ANGPTL4’ (≤170 ng/ml, serum ANGPTL4 within the normal range) and ‘high ANGPTL4’ (>170 ng/ml; serum ANGPTL4 more than 10% above normal range). We therefore considered whether having ‘high’ serum ANGPTL4 might be indicative of disease severity. No significant difference was found between ‘high’ and ‘low’ groups with respect to age, DAS score or CRP.

Given the stimulatory effect of ANGPTL4 on osteoclast activity, we also assessed possible correlations with the circulating marker of bone erosion, RANKL. Considering all patient groups, serum concentrations of RANKL ranged from 0–392.7 ng/ml, with most individuals having undetectable serum RANKL. To prevent skewing of the analysis by high value data points in a small sample size, we assessed this data according to whether patients exhibited detectable circulating RANKL (RANKL-positive or RANKL-negative). 85.7% of ‘high ANGPTL4’ RA patients were RANKL-positive, compared with only 16.7% of ‘low ANGPTL4’ RA patients (p<0.05) and 20.0% of controls (p<0.01) (Figure 4C).

## Discussion

Inhibition of osteoclast-mediated bone resorption is a crucial component of targeted strategies to alleviate bone destruction in RA. ANGPTL4, a hypoxia-induced adipokine, has previously been shown to stimulate osteoclast activity *in* *vitro*. This study demonstrates increased expression of ANGPTL4 within rheumatoid synovial tissue, as well as increased secretion of ANGPTL4 into the synovial fluid and serum of patients with RA.

This is the first description of ANGPTL4 expression in RA synovial tissue. We have previously reported that ANGPTL4 functions in a paracrine manner to stimulate osteoclast activity and induce bone resorption [13]. This effect was independent of RANKL, as ANGPTL4 prevented both loss of osteoclast viability and loss of bone resorption following RANKL inhibition with the soluble decoy receptor osteoprotegerin [13]. Expression of ANGPTL4 in bone-apposing resorbing osteoclasts, as well as strong expression in other cells of the rheumatoid synovium, could generate an environment that supports and promotes bone erosion. This makes it potentially attractive as a secondary target, outside the RANKL/RANK pathway, for strategies to specifically prevent osteolytic bone disease in RA [5].

ANGPTL4 also has the potential to contribute to cartilage matrix destruction. Multi-nucleated cells resorbing cartilage in RA have an osteoclast-like phenotype and human monocyte-derived osteoclasts are capable of cartilage matrix resorption *in* *vitro* [24]. RA articular chondrocytes over-express ANGPTL4 which, when induced by hypoxia *in* *vitro*, stimulates production of the matrix metalloproteinases MMP-1 and MMP-3 [21]. ANGPTL4 might therefore induce both bone and cartilage erosion in RA, via effects on MMP production and osteoclast activation, to increase joint destruction.

It is interesting to consider whether the 3 out of 8 RA cases seen to contain numerous osteoclasts is a typical ratio. While it is consistent with what we have previously observed [23], it is not possible to tell from the literature whether this is a typical proportion. Although many papers measure expression and/or secretion of osteoclast-associated factors, very few actually show pictures of osteoclasts in human RA and we have found no mention of the frequency with which osteoclast-rich RA occurs. Furthermore, due to the flattening of some osteoclasts if the synovium pulls away from the bone during tissue processing, clear images cannot always be obtained. However, regardless of the proportion of cases where osteoclasts can be observed by immunohistochemistry, osteoclast-mediated bone erosion is a common and characteristic feature of RA.

This is also the first description of HIF-1α expression in RA osteoclasts. HIF-1α stimulates increased osteoclast-mediated bone resorption under hypoxic conditions [14]. HIF-1α also controls the over-expression of VEGF-A by large osteoclasts containing >10 nuclei, as found in RA, versus small osteoclasts containing 2–5 nuclei, as found in OA [31]. As ANGPTL4 is also a HIF target gene, this observation might explain why we only detected ANGPTL4 expression in RA-, but not OA-, associated osteoclasts. Promotion of pathological bone resorption in RA might be a crucial component of the mechanism by which HIF drives disease progression [32].

Co-localisation of HIF-1α and ANGPTL4 in bone-apposing osteoclasts supports our previous *in* *vitro* observations that ANGPTL4 is induced by HIF-1α in human osteoclasts [13]. However, not all ANGPTL4-positive osteoclasts expressed HIF-1α. This might be due to cytokine-mediated effects on gene expression. In fibroblast-like synoviocytes from RA, for example, TNFα induced expression of HIF-1α but not ANGPTL4, whereas IL-4 induced ANGPTL4 but not HIF [22]. ANGPTL4 is additionally regulated by the PPAR family of transcription factors [33], which might influence expression patterns. All other cell types within the RA synovium that we found to express ANGPTL4 also expressed HIF-1α, both in this study and as reported previously [9], [12]. ANGPTL4 has been confirmed as a HIF target gene in human RA fibroblast-like synoviocytes *in* *vitro* [22].

ANGPTL4 mRNA and protein levels have previously been reported to increase in the early stages of murine collagen-induced arthritis, where expression was specific to the stromal fibroblast-like cells adjacent to blood vessels, suggestive of a role in angiogenesis [34] as reported in cancer [19], [20]. Human RA fibroblast-like synoviocytes exposed to hypoxia *in* *vitro* also exhibited increased ANGPTL4 mRNA and protein expression [22]. As well as confirming ANGPTL4 expression in RA synovial fibroblasts *in* *situ*, we have observed cytoplasmic expression of ANGPTL4 in many other cell types including, but not restricted to, CD68+ macrophages, plasma cells, endothelial cells and osteoclasts within the hyperplastic synovium. Such wide-spread induction could rapidly provide a large local pool of pro-angiogenic ANGPTL4 to also stimulate blood vessel formation in RA.

The concentration range of serum ANGPTL4 in our normal controls and patients with OA (11.9–154.7 ng/ml) agrees with the 2–158 ng/ml previously reported in the normal population [35]. Distinct sub-groups of ‘low ANGPTL4’ and ‘high ANGPTL4’ RA patients were apparent, having serum ANGPTL4 either within the normal range or at least 10% above this range (217.3–2235.0 ng/ml). If clinically or radiologically relevant information could be found to correlate with ‘high ANGPTL4’, serum level of ANGPTL4 may prove a useful prognostic or diagnostic tool.

In agreement with our hypothesis that ANGPTL4 contributes to bone erosions in RA, ‘high ANGPTL4’ patients were significantly more likely to have detectable concentrations of serum RANKL. Circulating RANKL has recently been described to correlate with the level of erosive damage in RA, as well as being a prognostic factor for the progression of this structural damage, as assessed by radiography over a 3 year period [36]. While we did not have sufficient numbers in the current study to replicate this data for ANGPTL4, the only two non-erosive patients in our study population exhibited two of the three lowest serum concentrations of ANGPTL4. Although a larger study cohort is required, with matched serum, synovial tissue/fluid and radiological data, it is interesting to speculate that ANGPTL4 might represent not only a serum marker of either progression of joint damage or response to treatment, but also a potential therapeutic target.

In summary ANGPTL4 is over-expressed in osteoclasts and other cells within the rheumatoid synovium, in the synovial fluid and in the serum of patients with RA. High serum ANGPTL4 in RA correlates with high circulating levels of RANKL. As ANGPTL4 stimulates osteoclast resorption activity *in* *vitro*, it may therefore mediate osteolytic erosion of bone and cartilage in RA, thereby contributing to joint destruction in addition to its established pro-angiogenic role [22], [34]. This suggests ANGPTL4 both as a possible target for therapies to treat several different aspects of the disease, as well as a novel marker for bone destruction in RA.
